# Prescription practices in the treatment of agitation in newly hospitalized Chinese schizophrenia patients: data from a non-interventional naturalistic study

**DOI:** 10.1186/s12888-019-2192-6

**Published:** 2019-07-10

**Authors:** Su-Zhen Zhang, Yong-Gang Mu, Qi Liu, Ying Shi, Li-Hua Guo, Ling-Zhi Li, Fu-De Yang, Yong Wang, Tao Li, Qi-Yi Mei, Hong-Bo He, Zhi-Yu Chen, Zhong-Hua Su, Tie-Bang Liu, Shi-Ping Xie, Qing-Rong Tan, Jin-Bei Zhang, Cong-Pei Zhang, Hong Sang, Wei-Feng Mi, Hong-Yan Zhang

**Affiliations:** 1 Department of Psychiatry, Huzhou 3rd Hospital, Huzhou, 313000 Zhejiang China; 2 Peking University Sixth Hospital, Peking University Institute of Mental Health, NHC Key Laboratory of Mental Health (Peking University), National Clinical Research Center for Mental Disorders (Peking University Sixth Hospital), 51, Huayuan Bei Road, Beijing, 100191 China; 3 Beijing HuiLongGuan Hospital, Huilong guan Changping District, Beijing, 100096 China; 4 Shanghai Mental Health Center, 600,Wanpingnan Street, Shanghai, 200030 China; 5 West China Hospital of Sichuan, 37, Guoxue Street, Wuhou District, Chengdu, 610041 Sichuan China; 6 Suzhou Guangji Hospital, 285, Guangji Street, Gusu District, Suzhou, 215008 Jiangsu China; 7 Guangzhou Psychiatric Hospital, 36, Mingxin Street, Liwan District, Guangzhou, 510000 Guangdong China; 8 The Seventh People’s Hospital of Hangzhou, 305, Tianmushan Street, Xihu District, Zhejiang, 310013 Hangzhou China; 9 Jining Mental Hospital, 1, Jidai Street, Jining, 272000 Shandong China; 10 Shenzhen Kangning Hospital, 1080,Cuizhu Street, Luohu District, Shenzhen, 518003 China; 11 Nanjing Brain Hospital, 264,Guangzhou Road, Gulou District, Nanjing, 210029 Jiangsu China; 12 Xijing Hospital of Forth Military Medical University, 15, Changlexi Road, Xi’an, 710032 Shanxi China; 13 The Third Affiliated Hospital of Sun Yat-sen University, 600, Tianhe Road, Tianhe District, Guangzhou, 510630 Guangdong China; 14 Harbin First Specific Hospital, 217, Hongwei Road, Harbin, 150056 Heilongjiang China; 15 Sixth Hospital of Changchun, 4596, Beihuan Road, Changchun, 130052 Jilin China

**Keywords:** Schizophrenia, Agitation, Prescription

## Abstract

**Background:**

Data on the pharmacological management of acute agitation in schizophrenia are scarce. The aim of this study is to investigate the prescription practices in the treatment of agitation in Chinese patients with schizophrenia.

**Methods:**

We conducted a large, multicenter, observational study in 14 psychiatry hospitals in China. Newly hospitalized schizophrenia patients with the PANSS-EC total score ≥ 14 and a value ≥4 on at least one of its five items were included in the study. Their drug treatments of the first 2 weeks in hospital were recorded by the researchers.

**Results:**

Eight hundred and 53 patients enrolled in and 847 (99.30%) completed the study. All participants were prescribed antipsychotics, 40 (4.72%) were prescribed benzodiazepine in conjunction with antipsychotics and 81 were treated with modified electric convulsive therapy (MECT). Four hundred and 12 (48.64%) patients were prescribed only one antipsychotic, in the order of olanzapine (120 patients, 29.13%), followed by risperidone (101 patients, 24.51%) and clozapine (41 patients, 9.95%). About 435 (51.36%) participants received antipsychotic polypharmacy, mostly haloperidol + risperidone (23.45%), haloperidol+ olanzapine (17.01%), olanzapine+ ziprasidone (5.30%), haloperidol + clozapine (4.37%) and haloperidol + quetiapine (3.90%). Binary logistic regression analysis suggests that a high BARS score (OR 2.091, 95%CI 1.140–3.124), severe agitation (OR 1.846, 95%CL 1.266–2.693), unemployment or retirement (OR 1.614, 95%CL 1.189–2.190) and aggressiveness on baseline (OR 1.469, 95%CL 1.032–2.091) were related to an increased antipsychotic polypharmacy odds. Male sex (OR 0.592, 95%CL 0.436–0.803) and schizophrenia in older persons (age ≥ 55 years, OR 0.466, 95%CL 0.240–0.902) were less likely to be associated with antipsychotic polypharmacy.

**Conclusion:**

The present study demonstrates that monotherapy and polypharmacy display equally common patterns of antipsychotic usage in managing agitation associated with schizophrenia in China. The extent and behavioral activities of agitation and several other factors were associated with polypharmacy.

**Electronic supplementary material:**

The online version of this article (10.1186/s12888-019-2192-6) contains supplementary material, which is available to authorized users.

## Background

Agitation among psychiatric inpatients, particularly those with schizophrenia or bipolar disorder, is a major public health concern. It can rapidly escalate to aggression and violence and poses a considerable risk to the patients themselves and to others [1, 2]. Recent studies indicate that agitation significantly contributes to rising medical costs and caregiver burden [3, 4]. In the United States alone, agitated patients with schizophrenia accounted for 900,000 annual visits to psychiatric emergency services, representing 21% of all psychiatric emergency visits [5]. According to the State of Acute Agitation at Psychiatric Emergencies in Europe (STAGE) study, schizophrenia was the underlying in 47.27% (78 of 165) of psychiatric agitation episodes [6]. In China, we previously screened 1400 newly hospitalized patients with schizophrenia, finding that 60.92% (853 of 1400) were agitated [7]. Current psychiatric recommendations on agitation promote the early use of verbal de-escalation techniques [2, 8]. However, a considerable percentage of schizophrenia patients with agitation still require psychopharmacological approaches, including typical or first-generation antipsychotic drugs, atypical or second-generation antipsychotic drugs, and benzodiazepines [9, 10]. Depending on the patient’s level of cooperation or the perceived onset of the drug effect, there are four routes available in clinical practice: inhalation, oral administration, intramuscular injection, and intravenous injection. Therefore, an array of pharmacological options administered via different routes exists, providing physicians with numerous treatment alternatives.

Typical antipsychotics have proven effectiveness in the treatment of agitation, particularly haloperidol [10]. However, considering the high risk of extrapyramidal side effects and their superior efficacy and lower adverse effect burden, atypical antipsychotics are highly preferred in clinical practice [8, 9]. Although monotherapy is recommended in clinical practice guidelines for the use of antipsychotics in the management of schizophrenia [11, 12], polypharmacy is common, ranging from 20.4 to 69.6% across different countries [13–16]. In a 2002 study investigating the factors influencing the prescription of antipsychotics to patients with schizophrenia in China, about 25.9% of 3523 patients received polypharmacy [17]. In Hong Kong, a survey investigated the current prescribing patterns of agitation in emergency departments for patients without a specific diagnosis on record [18]. There are no published data on prescription practices for schizophrenia with agitation in China. Thus, the aim of our study was to reveal the prescription practices in the treatment of agitation in Chinese patients with schizophrenia.

## Methods

### Study design and subjects

This study was part of the Investigation of Prevalence and Treatment of Agitation in Newly Hospitalized Schizophrenia in China (IPTASC, Hongyan Zhang Group Investigators).

This observational, multicenter, cross-sectional study is aimed to explore the patterns of antipsychotic use for agitation among newly hospitalized Chinese schizophrenia patients and to the factors possibly associated with the prescription pattern. Data specific to this report were collected between June 2014 to December 2014. All of the patients in this study were consecutively recruited from 14 psychiatric hospitals in representative regions of China (see the author affiliations). The study was conducted after obtaining approval from the ethics committees of Peking University Sixth Hospital and all other participating hospitals. All participants provided written informed consent from the patients themselves or their legal guardians (if involuntary admission) prior to the enrollment.

Inclusion criteria were: age ≥ 18 years; Han ethnicity; newly hospitalized (admission within 24 h) in one of the 14 hospitals; diagnosis of schizophrenia or hallucinatory paranoid state based on the International Statistical Classification of Diseases and Related Health Problems, 10th revision (ICD-10); Clinical Global Impression-Severity (CGI-S) score ≥ 4, and Positive and Negative Syndrome Scale–Excited Component (PANSS-EC) score ≥ 14 and at least one of its five items was ≥4. Exclusion criteria were: had comorbidity that caused agitation, such as substances use disorder, central nervous system disease, including Parkinson’s disease, Alzheimer’s disease, other types of dementia, encephalitis and meningitis and general medical conditions (e.g., thyrotoxicosis, hypoglycemia, brain traumas.); incapable of completing the survey because of mental or physical dysfunction were also excluded; non-schizophrenia patients; withdrew informed consent.

Data on antipsychotic patterns and potential related factors were obtained from the patients and their caregivers through a specially designed questionnaire. The questionnaire includes basic general demographics, clinical characteristics including the course of schizophrenia, age of onset, number of lifetime hospitalizations, admission patterns (whether voluntary hospitalization or not), CGI-S scores, BARS scores, PANSS-EC scores, and MOAS scores and treatment information. After admission, the first two parts would be completed in 24 h by his/her psychiatrist. Two weeks later, the same psychiatrist re-diagnosed the patients to exclude any non-schizophrenic patients and collect the treatment information. A brief enrollment IPTASC study is presented in Additional file 1: Figure S1.

### Statistical analysis

After the surveys were completed, all questionnaires were sent to Peking University Sixth Hospital for data analysis. The copyright of the dataset belongs to Peking University Sixth Hospital. Differences in demographics, clinical characteristics, and possible associated factors were calculated between the antipsychotic monotherapy group and the polypharmacy group. We adopted the t test and the χ2 test to examine theses factors in relation to antipsychotic prescription practices. Factors that were significantly different between groups were analyzed using a binary logistic regression model. For all statistical analyses, differences were considered significant at a two-tailed *p* < 0.05. All statistical analyses were performed with SPSS 16.0 software (SPSS, Chicago, IL).

## Results

### Demographic and clinical characteristics

Of 1512 patients screened, we enrolled 853 patients who met the inclusion criterion of agitation. Because 6 individuals did not provide prescription information, 847 participants (99.30%; male, 51.00%) were included in the final analysis. Among them, 435 (51.36%) were subject to polypharmacy during the first 2 weeks. The demographic and disease characteristics of the participants, along with those of the polypharmacy and monotherapy groups, are presented in Table 1. Mean age was 34.62 ± 11.79 years. Most of the participants (70.84%) had less than 12 years of education (education level lower than college) and 36.48% were married. Approximately three-quarters (77.57%) were involuntarily hospitalized and the mean lifetime number of hospitalizations was 1.96 ± 3.10. About half (51.36%) of the participants were employed and the mean duration of schizophrenia was 8.20 ± 8.80 years. Approximately 47.46% of the participants had severe agitation and 70.84% had aggressive behavior(s) at baseline (Table 1).

**Table 1:** Demographic and clinical characteristics of respondent patients

	Overall (*n* = 847)	Monotherapy (*n* = 412)	Polypharmacy (*n* = 435)	χ^2^/t	*p*
Sex, *n*(%)^a^					
Male	432 (51.00)	234	198	9.97	0.002
Female	409 (48.29)	177	232		
Age, mean (SD), y	34.62(11.79)	35.38(12.52)	33.92(11.02)	1.78	0.075*
Schizophrenia in older persons, n(%) ^b^					
Yes	51 (6.02%)	34	17	7.23	0.007
No	796(93.98%)	368	411		
Educational level, n(%) ^c^					
≤ High school	600 (70.84)	288	312	0.40	0.526
≥ College	242(28.57)	122	120		
Marital status, n(%)^d^					
Married	309(36.48)	142	167	1.64	0.65
Single	444(52.42)	225	219		
Divorced	79(9.33)	38	41		
Widowed	10(1.18)	5	5		
Living alone, n(%)^e^					
Yes	40(4.72)	28	12	7.12	0.005
No	802(94.69)	381	421		
Employment, n(%)^f^					
Employed	435(52.76)	187	248	11.06	0.001
Unemployed or retired	400(47.23)	218	182		
Health insurance ^g^					
Yes	297(35.06)	137	160	0.94	0.332
No	542(63.99)	269	273		
Admission pattern ^h^					
Voluntary hospitalization	179(21.13)	65	114	14.23	1.62E-4
Involuntary hospitalization	657(77.57)	343	314		
Age at onset, mean (SD), y	26.53(9.66)	27.15(10.34)	25.94(8.88)	1.81	0.071*
Course of schizophrenia, mean (SD), y	8.20(8.80)	8.39(8.95)	8.03(8.01)	0.58	0.566*
Number of hospitalizations, mean (SD)	1.96(3.10)	1.95(3.09)	1.97(3.11)	−0.09	0.932*
CGI score, mean (SD)	5.38(0.81)	5.38(0.77)	5.38(0.84)	0.12	0.908*
BARS score, mean (SD)	5.43(1.19)	5.20(1.14)	5.65(1.18)	−5.60	2.87E-8
Antipsychotics used in the previous week, n(%)^i^					
Yes	350(41.32)	163	187	2.99	0.084
No	465(54.90)	245	220		
Aggressiveness on baseline (MOAS ≥ 4), *n*(%)					
Yes	600(70.84%)	273	327	11.58	0.001
No	247(29.16)	135	95		
Severe agitation					
Yes	402(47.46%)	161	241	22.61	1.98E-6
No	445(52.54%)	251	194		

Notes: ^a^ Six participants (0.71%) did not provide this information. ^b^ Seventeen participants (2.01%) did not provide this information. ^c^ Five participants (0.6%) did not provide this information. ^d^ Five participants (0.59%) did not provide this information. ^e^ Five participants (0.59%) did not provide this information. ^f^ Twelve participants (1.42%) did not provide this information. ^g^ Eight participants (0.94%) did not provide this information. ^h^ Eleven participants (1.30%) did not provide this information. ^i^ Thirty two participants (3.79%) did not provide this information.* T test

CGI Clinical Global Impressions-Severity, BARS Behavioral Activity Rating Scale, MOAS Modified Overt Aggression Scale

### Prescription patterns and frequency of antipsychotic medications

All participants were prescribed antipsychotics. Also, 40 (4.72%) were prescribed benzodiazepine in conjunction with antipsychotics and 81 were treated with modified electroconvulsive therapy (MECT). The most frequently selected route of administration was oral alone, accounting for over half of the 847 prescriptions. The next most common administration route was a combination of oral and intramuscular injections (39.91% of prescriptions). Only 47 patients (5.55%) received intramuscular drug injection alone (Table 2). None took antipsychotics through intravenous administration. Among 847 patients, only 2 were orally administered haloperidol, with 99.33% (300 of the 302 patients) intramuscularly administered the drug.

**Table 2:** Prescription patterns and administration routes of psychotic medications for respondent patients

	Overall (*n* = 847)	Schizophrenia In older persons (*n* = 51)
Prescription patterns, *n*(%)		
Monotherapy	412(48.64)	34(66.67)
Typical antipsychotic only	41(4.84)	5(9.80)
Atypical antipsychotic only	371(43.80)	29(56.86)
Polypharmacy	435(51.36)	17(33.33)
Typical+ Typical	3(0.35)	0
Typical+ Atypical	287(33.88)	13 (25.49)
Atypical+ Atypical	145(17.12)	4 (284.31)
Concomitant medications, *n*(%)		
Benzodiazepines	40(4.72%)	2(3.92)
MECT, *n*(%)	81(9.56%)	2(3.92)
Administration route, *n*(%)		
Oral route alone	462(54.55%)	30(58.82)
A combination of oral and intramuscular injections route	338(39.91%)	14(27.45)
Intramuscular injections route alone	47 (5.55%)	7(13.73)

As shown in Tables 2, 412 patients (48.64%) were prescribed only one antipsychotic (either in single or multiple formulations), including 371 patients who were prescribed a second-generation antipsychotic. The most commonly prescribed agents were olanzapine (120, 29.13%) in the monotherapy group, risperidone (101, 24.51%), and clozapine (41, 9.95%) (Table 3).

**Table 3:** The five most common used psychotic medications for respondent patients

Total (*n* = 847)		Monotherapy (*n* = 412)		Polypharmacy (*n* = 435)	
Medication, *n*(%)		Medication, *n*(%)		Medication, *n*(%)	
Haloperidol	302(35.66)	Oanzapine	120(29.13)	Haloperidol+ Risperidone	102(23.45)
Olanzapine	275(32.47)	Risperidone	101(24.51)	Haloperidol+ Olanzapine	74(17.01)
Risperidone	259(30.58)	Clozapine	41(9.95)	Olanzapine+ Ziprasidone	23(5.29)
Clozapine	125(14.76)	Quetiapine	36(8.74)	Haloperidol+ Cozapine	19 (4.37)
Ziprasidone	105(12.40)	Haloperidol	28(6.80)	Haloperidol+ Quetiapine	17 (3.90)

About half of the participants (435, 51.36%) were treated with antipsychotic polypharmacy, with 396 (46.57%) receiving two different antipsychotics, 38 (4.49%) receiving three different antipsychotics, and 1 (0.12%) receiving four different antipsychotics. The frequencies of multiple antipsychotics were in the following order: haloperidol + risperidone (23.45%), haloperidol + olanzapine (17.01%), olanzapine + ziprasidone (5.30%), haloperidol + clozapine (4.37%), and haloperidol + quetiapine (3.90%). There were significant differences between the polypharmacy and monotherapy groups in sex, age, admission pattern, living alone, aggressiveness at baseline, severe agitation, and BARS score (*p* < 0.01, Table 1).

### Characteristics associated with antipsychotic polypharmacy

The results of binary logistic regression analysis are shown in Table 4. A high BARS score (odds ratio [OR] 2.091, 95% confidence interval [95%CI] 1.1400–3.124), severe agitation (OR 1.846, 95%CI 1.266–2.693), unemployment or retirement (OR 1.614, 95%CI 1.189–2.190), and aggressiveness at baseline (OR 1.469, 95%CI 1.032–2.091) were suggested to be related to an increased antipsychotic polypharmacy odds, whereas male sex (OR 0.592, 95%CI 0.436–0.803) and schizophrenia in older persons (age ≥ 55 years, OR 0.466, 95%CI 0.240–0.902) were less likely to be associated with antipsychotic polypharmacy.

**Table 4:** Characteristics associated with antipsychotic polypharmacy for respondent patients

	OR	95%CI	*P*
BARS score	2.091	1.140–3.124	3.16E-4
Severe agitation	1.846	1.266–2.693	0.001
Unemployed or retired	1.614	1.189–2.190	0.002
Aggressiveness on baseline (MOAS≥4)	1.469	1.032–2.091	0.033
Schizophrenia in older persons	0.466	0.240–0.902	0.023
Living alone	0.438	0.209–0.919	0.029
Male sex	0.592	0.436–0.803	0.001

## Discussion

To our knowledge, this is the first report of prescription patterns among schizophrenia patients with agitation in China. The current expert guidelines and research data [9, 10, 19, 20] indicate that, in the management of agitation due to a psychiatric illness, second-generation antipsychotics (risperidone, olanzapine, ziprasidone) are to be selected as first-line agent. In our study, most patients (94.81%) received atypical antipsychotics. The widespread use of second-generation antipsychotics may be due to their well-known superiority in terms of adverse effects. Specifically, the most used antipsychotic was haloperidol, identified in 302 patients (35.66%), followed by olanzapine in 276 (32.59%) and risperidone in 258 (30.46%). Given its easy accessibility and its long track record of efficacy in the management of agitation, haloperidol is an important treatment choice in cases of limited resources. Previous studies reported economic status as a major factor in antipsychotic prescription in Chinese outpatients with schizophrenia [17]. Consequently, the lower price of haloperidol may also have contributed to its popularity.

The American Association for Emergency Psychiatry (AAEP) and World Federation of Societies of Biological Psychiatry (WFSBP) consensus guidelines recommend oral antipsychotics over intramuscular injections if possible [9, 12]. Our study found that 385 of 847 patients (45.5%) received intramuscular injections. An unstable course of illness, irritability, and uncooperativeness promote the use of intramuscular injections in agitation management. Additionally, intramuscular administration is preferred by clinicians for rapid and effective treatment. Note that a systematic review of studies from 2001 to 2010 showed that oral and intramuscular medications are equally effective in rapidly reducing psychotic agitation in the emergency department [21]. Nonetheless, further studies are needed.

Approximately 51.36% of patients were prescribed antipsychotic polypharmacy. Our rate of polypharmacy is much higher than the rate from a 2011 report on schizophrenia in China in both inpatients and outpatients [17] and the rate from a 2015 report in a Hong Kong emergency department [18]. There are two possible reasons for this difference: (1) newly hospitalized patients with agitation usually have more severe symptoms, increasing the difficulty of treatment; and (2) the desire to enhance or accelerate treatment efficacy [22] or to reduce the dose of antipsychotics with certain adverse effects [23, 24].

The key factors associated with polypharmacy in the present study were a high BARS score (OR 2.091, 95%CI 1.1400–3.124) and severe agitation (OR 1.846, 95%CI 1.266–2.693). The seven-point BARS assesses behavioral activities in agitated patients with high reliability and convenience, with results ranging from sedation, to normal activity, to agitation [25]. Agitation is an established short-term factor associated with physical aggression [26]. The assessment of behavioral activities in agitated patients at an early stage may help to identify the risk of aggression and suggest specific treatment interventions. According to a recent survey of 583 community-dwelling patients with schizophrenia or bipolar disorder, the most common experience of agitation was in the form of internal feelings, including uneasiness, restlessness, and nervousness [27]. A qualitative study with healthcare professionals defined three states of agitation, with aggression or violence only included in severe agitation [28]. Better assessment and effective management of severe agitation could potentially avoid aggressive episodes. In agreement with this, several studies and published consensus guidelines have focused on acute episodes of severe agitation [29–31]. We also found that unemployment or retirement (OR 1.614, 95%CI 1.189–2.190) and aggressiveness at baseline (OR 1.469, 95%CI 1.032–2.091) were associated with polypharmacy. Unemployment may be a sign of disease severity.

In contrast with previous research [32, 33], we observed a negative association between polypharmacy and male sex (OR 0.592, 95%CI 0.436–0.803). In a study of 43 severely agitated patients with schizophrenia spectrum disorders, there was a sex effect in the poor treatment response in women [30]. Furthermore, some studies reported that women committed more physical assaults than men during the 4-week inpatient treatment [34, 35]. These two findings could imply a tendency toward polypharmacy in women. Our findings are consistent with existing research that older people with schizophrenia (age ≥ 55 years) are less likely to receive antipsychotic polypharmacy [32, 33]. In contrast to younger schizophrenia patients, older patients are more prone to adverse effects, including extrapyramidal side effects and metabolic syndrome [36]. It is advisable to start with a single, low initial dose and titrate slowly. A study comprising 8792 elderly schizophrenia patients suggested that the combined use of first- and second-generation antipsychotics was positively associated with relapse (OR 1.70, 95%CI 1.25–2.31) [37]. Besides the consideration of safety, older patients are less prone to aggressive episodes [38].

### Limitations

There are some limitations to this study. First, we lack data on the use of non-pharmacological interventions (verbal de-escalation, physical restraint, and seclusion) to calm agitated patients, which may have some interactions with the prescription pattern. Thus, limited data are available to provide insight into the relationship between non-pharmacological and pharmacological interventions. Second, there is potential for a discrepancy between prescribing decisions in practice. The reasons for the clinicians’ prescribing decision processes and patients’ preferences for prescriptions were not explored. The confounding effects of these differences could not thus be addressed. Third, the dose of each drug was not taken into consideration, mainly because the prescriptions of newly hospitalized agitated schizophrenia patients are not stable in the first 2 weeks. Fourth, legal status or past history of crime was not measured.

## Conclusions

With a large sample size and a large number of participating sites involved in a standardized assessment, our findings have some important clinical implications. The present study demonstrates that monotherapy and polypharmacy are equally common in antipsychotic application in managing agitation associated with schizophrenia in China. The extent and behavioral activities of agitation and several other factors were associated with polypharmacy. Additional factors should be evaluated in future studies.

## Additional file

Additional file 1: **Figure S1.** Enrollment IPTASC Study. (PDF 22 kb)

## Data Availability

The datasets used during the current study are available from the corresponding author on reasonable request.
