# Supplementary figures and images for: Prescription practices in the treatment of agitation in newly hospitalized Chinese schizophrenia patients: data from a non-interventional naturalistic study
Source: BMC Psychiatry. 2019 Jul 10;19:216. doi: 10.1186/s12888-019-2192-6 (PMC6617899; doi:10.1186/s12888-019-2192-6)

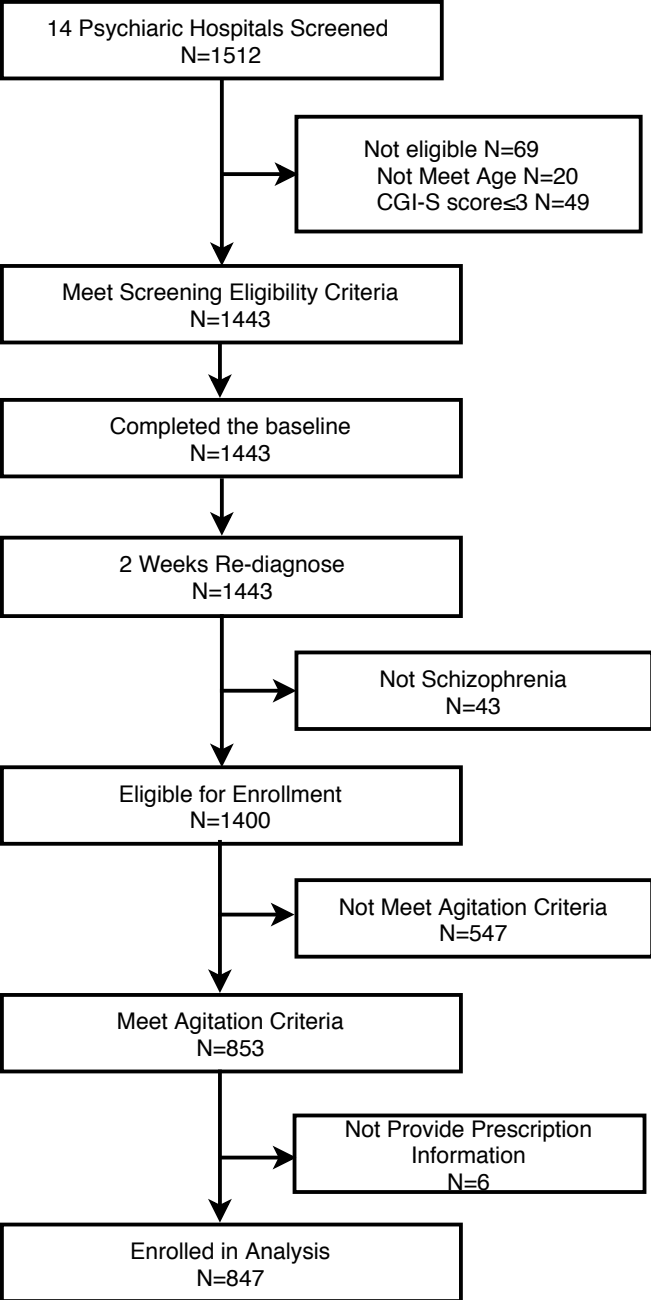

Supplement: Supplementary file 1 — Figure S1. Enrollment IPTASC Study. (PDF 22 kb) [file 12888_2019_2192_MOESM1_ESM.pdf]
